# Physical education policy compliance and Latino children’s fitness: Does the association vary by school neighborhood socioeconomic advantage?

**DOI:** 10.1371/journal.pone.0178980

**Published:** 2017-06-07

**Authors:** Emma V. Sanchez-Vaznaugh, Lisa Goldman Rosas, José Ramón Fernández-Peña, Jonggyu Baek, Susan Egerter, Brisa N. Sánchez

**Affiliations:** 1 Health Education Department and Health Equity Institute, San Francisco State University; Center on Social Disparities in Health, University of California San Francisco, San Francisco, California, United States of America; 2 Family Community Medicine, Center on Social Disparities in Health, University of California San Francisco School of Medicine, San Francisco, California, United States of America; 3 Stanford Prevention Research Center, Program on Prevention Outcomes and Practices, Palo Alto, California, United States of America; 4 Center for Social Epidemiology and Population Health, University of Michigan, Ann Arbor, United States of America; 5 Department of Biostatistics, School of Public Health, University of Michigan, Ann Arbor, United States of America; Hunter College, UNITED STATES

## Abstract

**Objectives:**

To investigate the contribution of school neighborhood socioeconomic advantage to the association between school-district physical education policy compliance in California public schools and Latino students’ physical fitness.

**Methods:**

Cross-sectional Fitnessgram data for public-school students were linked with school- and district-level information, district-level physical education policy compliance from 2004–2005 and 2005–2006, and 2000 United States Census data. Multilevel logistic regression models examined whether income and education levels in school neighborhoods moderated the effects of district-level physical education policy compliance on Latino fifth-graders’ fitness levels.

**Results:**

Physical education compliance data were available for 48 California school districts, which included 64,073 Latino fifth-graders. Fewer than half (23, or 46%) of these districts were found to be in compliance, and only 16% of Latino fifth-graders attended schools in compliant districts. Overall, there was a positive association between district compliance with physical education policy and fitness (OR, 95%CI: 1.38, 1.07, 1.78) adjusted for covariates. There was no significant interaction between school neighborhood socioeconomic advantage and physical education policy compliance (p>.05): there was a positive pattern in the association between school district compliance with physical education policy and student fitness levels across levels of socioeconomic advantage, though the association was not always significant.

**Conclusions:**

Across neighborhoods with varying levels of socioeconomic advantage, increasing physical education policy compliance in elementary schools may be an effective strategy for improving fitness among Latino children.

## Introduction

For nearly two decades, there has been strong interest in environmental and policy interventions that promote healthy eating, physical activity and healthy weight, particularly in school settings[1–4]. The National Academy of Sciences and the American Heart Association recommended that schools play a central role to prevent childhood obesity by increasing physical activity and fitness levels through different strategies including physical education[2,3]. School based physical education, including physical education policy and related compliance have been associated with greater levels of children’s physical activity and fitness [5–8]. Although physical education policies may have the greatest impact on the amount of time U.S. youth engage in moderate to vigorous physical activity [9] compliance with such policies may also play an important role [7,10,11].

School-based physical education policies maybe particularly important for subgroups of children such as Latinos who have limited opportunities to participate in extracurricular activities that promote physical activity. Relative to non-Hispanic white children, Latino children are less likely to meet national guidelines for physical activity [12–14] and are more likely to be physically unfit [15]. Nationally, Latino children have the highest prevalence of obesity [16]. These children are also more likely to have limited venues for physical activity [17,18] and to attend schools in neighborhoods with fewer socioeconomic resources. The socioeconomic characteristics of schools and their surrounding neighborhoods may either enhance or undermine the influence of compliance with physical education policy (hereafter “PE”) on children’s fitness. Studies have found that socioeconomically advantaged schools were more likely to have a trained PE teacher and to have smaller PE classes,[19] and that students in affluent schools had more opportunities for physical activity than those in less socioeconomically advantaged schools [20]. Schools located in socioeconomically advantaged neighborhoods may have resources to offer students more diverse programs, increasing opportunities for regular physical activity and improved fitness. These prior studies raise the possibility that school-based physical education policies may play an important role in promoting physical fitness among Latino children, particularly those who attend schools in socioeconomically disadvantaged neighborhoods. Latinos are the youngest and fastest growing US population subgroup, and are likely to significantly contribute to future US population health trends. Thus, identifying effective policy and environmental strategies are needed to reduce obesity among Latino children through physical activity and fitness; yet, there is limited researched in this area [21]. In this study, we examine the possible combined influences of school district compliance with physical education policies and school neighborhood socioeconomic advantage on fitness levels among Latino children in California. California offers a unique opportunity to empirically evaluate the joint influences of compliance with PE policy and school neighborhood socioeconomic advantage for several reasons. California law requires schools to provide students in grades 1–6 with a minimum of 200 minutes of PE every ten days [22]. Since 2002, state law has also mandated that the California Department of Education (CDE) monitor compliance with this standard. Moreover, data from the California Physical Fitness Test (the ‘Fitnessgram’) are available for public school students in California through the CDE and can be linked with district-level data on compliance with state PE policy.

## Methods

### Data sources

#### School district-level data

Information on compliance with PE policy during the academic years 2004–2005 and 2005–2006 was obtained through a Freedom of Information Act request to the CDE [23], which oversees and collects compliance data through Categorical Programs Monitoring (CPM) [24,25]. Each of the 1,043 California school districts is assigned to one of four monitoring cycles, making each district eligible for monitoring once every four years; 10% of eligible districts are randomly selected for review each year, and the state’s Superintendent of Public Instruction Education may select additional districts for review as well. During the two school years studied here (2004–2005 and 2005–2006), data on district-level compliance were available for 55 (5.7%) of California school districts; CDE provided the names of these districts and whether they had complied with the state PE policy.

#### School-level data

We obtained school-level data, including school addresses, from publicly-available CDE databases. Geo-coded school addresses provided census-tract level socioeconomic information from the 2000 US Census, allowing us to examine levels of income and educational attainment for residents of school neighborhoods.

#### Student-level data

Student-level information was obtained from the California Physical Fitness Test (‘Fitnessgram’). Collected annually at schools since 1999, Fitnessgram data include information on age, sex, race/ethnicity, school attended, grade, measures of body composition, and physical fitness test scores for all fifth-, seventh-, and ninth-grade students statewide. This study used Fitnessgram data for Latino students collected during the 2004–2005 and 2005–2006 school years, corresponding to the period for which school district-level physical education policy compliance data were available for this study. Because physical activity behaviors are established early in life [26–28], and elementary-aged children are particularly vulnerable because they receive the shortest amount of mandated time in active PE in California[29], this study focused on the subset of fifth grade students.

Study analyses were restricted to districts that had compliance data and at least one Latino student (i.e., 48 of the 55 with compliance data). Of the 68,741 Latino fifth-graders attending schools in these 48 districts, 4,668 (6.8%) were excluded because key data, such as fitness information, were missing. Excluded students did not differ from included children with respect to other available individual-level factors such as sex and age. The analytic dataset comprised 64,073 fifth-grade Latino students attending 852 schools in 48 districts statewide.

This study was exempted from full IRB review from the author’s academic institutions, since the study involved secondary data with no individual identifiers.

### Study variables

#### Physical fitness

Student fitness levels were defined based on a student’s performance in the 1-mile run or walk test, as recorded in Fitnessgram. Standards for healthy fitness zones created by The Cooper Institute [30] were used to categorize students into groups consistent with CDE classifications: “needs improvement,” “meets the desired performance goal,” or “exceeds the desired performance goal.” For example, the cut-off for meeting the performance goal for the one-mile walk or run among ten-year-old fifth-grade girls was 12 minutes/30 seconds and 11 minutes/30 seconds for boys. Students that completed the test before the cut-off time were classified as exceeding the performance goal; students who took longer were classified as needing improvement. We examined fitness as a dichotomous outcome, comparing students who met or exceeded standards with those classified as needing improvement.

#### District-level compliance with PE policies

Districts were classified as either compliant or noncompliant with the required provision of at least 200 minutes of physical education to elementary school children every ten days, based on data from CDE reports for each of the 48 school districts described above. School districts reported if elementary students (grades 1–6) in every district school received physical education instruction for a minimum of 200 minutes every 10 school days throughout the school year [25]. The CDE determines whether each monitored district was in compliance with the required physical PE provisions based on reviews of district data and documents provided by the district to CDE, data screens, and interviews and direct observations during on-site visits to a randomly-selected subset of schools. If any school in a given district is found during an on-site visit to be out of compliance with PE policies, CDE considers the entire district to be noncompliant [25].

#### School-level neighborhood socioeconomic resources

The level of socioeconomic resources in each school’s surrounding neighborhood was characterized using two variables based on Census data: (a) neighborhood *income level*, measured as the annual median household income in the census tract in which the school was located; and (b) neighborhood *educational attainment*, measured as the proportion of census tract residents ages 25 and older who had completed college (i.e., with 16 or more years of schooling).

#### Other variables

Additional district-level characteristics included the number of schools, total student enrollment, and percent of children eligible for free or reduced-price meals in the district. School-level variables included total school enrollment, the proportion of students who were Latinos as a measure of Latino segregation [31], and the proportion of students eligible for free or reduced-price meals. Other student-level variables included age and gender.

This research was exempt from IRB review because we used secondary data.

### Statistical analyses

Characteristics of districts, schools, and students in the study population were assessed overall and by district-level compliance with PE policies, using the R statistical package [32]. Multilevel logistic regression models were constructed to estimate odds ratios describing the relative likelihood of physical fitness among Latino fifth-grade students in PE policy-compliant vs. noncompliant school districts, while accounting for ‘nesting’ of students within schools and schools within districts. To formally examine whether the association between district-level PE policy compliance and fitness among Latino fifth-graders differed depending on income and education levels in school neighborhoods, an interaction term was included in a model adjusting for student-, school-, and district-level characteristics. School-level covariates were categorized into quartiles because exploratory analyses revealed non-linear (quadratic) associations with fitness. Each district-level covariate was included as an indicator variable denoting whether the measure was above or below the median value for all 48 districts.

## Results

The study included 64,073 Latino fifth-graders attending 852 schools in the 48 districts for which compliance data were available. Fewer than half (23, or 46%) of districts were found to be in compliance with state PE policies. Most schools (677, or 80%) were located in noncompliant districts, and the majority of Latino fifth-graders (84%) in the study attended schools in noncompliant districts.

School neighborhood income and education levels, Latino student enrollment, and school-level proportion of Latino students were each significantly associated with PE policy compliance (Table 1). Compared with schools in noncompliant districts, those in PE policy compliant districts were: less likely to be located in low-income neighborhoods; more likely to be located in neighborhoods with lower proportions of college-educated residents; and less likely to have higher proportions of Latino students.

**Table 1:** Characteristics of schools^a^ in California school districts with available compliance^b^ data; years 2004–2005 and 2005–2006.

	Was school located in a district in compliance^b^ with state physical education policies?		
	Yes (n = 175)	No (n = 677)	p-value^c^
**School characteristics**			
Total school enrollment			0.008
< 504	20.0	26.0	
≥ 504 and < 680	27.4	24.5	
≥ 680 and < 881.5	39.4	21.4	
≥ 881.5	13.1	28.1	
% of enrolled students who were Latino			0.011
< 44.8	33.7	22.9	
≥ 44.8 and < 67.3	20.0	25.8	
≥ 67.3 and < 86.5	30.9	23.8	
≥ 86.5	15.4	27.5	
% of enrolled students eligible or received free/reduced priced meals			0.070
< 61.2	33.1	22.7	
≥ 61.2 and < 81.3	33.7	22.9	
≥ 81.3 and < 92.1	22.3	25.7	
≥ 92.1	10.9	28.7	
**School neighborhood**^d^ **characteristics**			<0.001
Median annual household income			
< $ 28,934	12.0	28.4	
≥ $ 28,934 and < $ 37,561	33.1	22.9	
≥ $ 37,561 and < $ 49,330	29.1	23.9	
≥ $ 49,330	25.7	24.8	
% of residents with 16 or more years of completed education			0.023
<6.3	23.4	25.6	
≥ 6.3 and < 12.2	37.1	21.4	
≥ 12.2 and < 24.3	20.0	26.6	
≥ 24.3	19.4	26.4	

^a^Schools were excluded if all students in the school had missing physical fitness data.

^b^Compliance defined as providing the minimum requirement for elementary-school students of 200 minutes of physical education every 10 days.

^c^ *p*-values testing association between compliance and quartiles of school characteristics.

^d^Refers to the census tract in which school is located.

Source: Authors' analysis of data from the Census 2000 Summary File 3 and from the CBEDS School Information Form and California School Free or Reduced Meal Program (both publicly available on the CDE website (http://www.cde.ca.gov/ds/sd/sd/ and http://www.cde.ca.gov/ds/sh/cw/filesafdc.asp).

Overall, forty-four percent of Latino fifth-graders did not meet physical fitness standards. Latino students in policy compliant districts were younger, more likely to be girls, and less likely to need improvement for fitness standards than their counterparts in non-compliant districts (Table 2).

**Table 2:** Characteristics of Latino 5^th^-graders in California school districts with available compliance^a^ data (2004–2005 and 2005–2006).

		Did student attend school in a district in compliance^b^ with state physical education policies?		
	Total sample (N = 64,073)	Yes (N = 10,250)	No (N = 53,823)	
Characteristics	Percent	Percent	Percent	P-values for difference^b^
**Age at last birthday, in years**				
10	51.9	53.2	51.6	0.0003
11	42.9	42.2	43.0	
12+	5.3	4.6	5.4	
**Gender**				
Male	50.6	49.6	50.8	0.0187
Female	49.4	50.4	49.2	
**Fitness**				
Needs improvement	44.1	42.2	44.4	< .0001
Meets standard	41.2	41.1	41.3	
Exceeds standard	14.7	16.7	14.3	

^a^Compliance measured at the district level and defined as providing the minimum requirement for elementary-school students of 200 minutes of physical education every 10 days.

^b^ *p*-values based on the Kolmogorov–Smirnov test comparing both the location and shape of the distributions for compliant and non-compliant school districts.

*Source*: Authors' analysis of data from the California Fitnessgram (2004–2006), California Department of Education.

**Fig 1:**
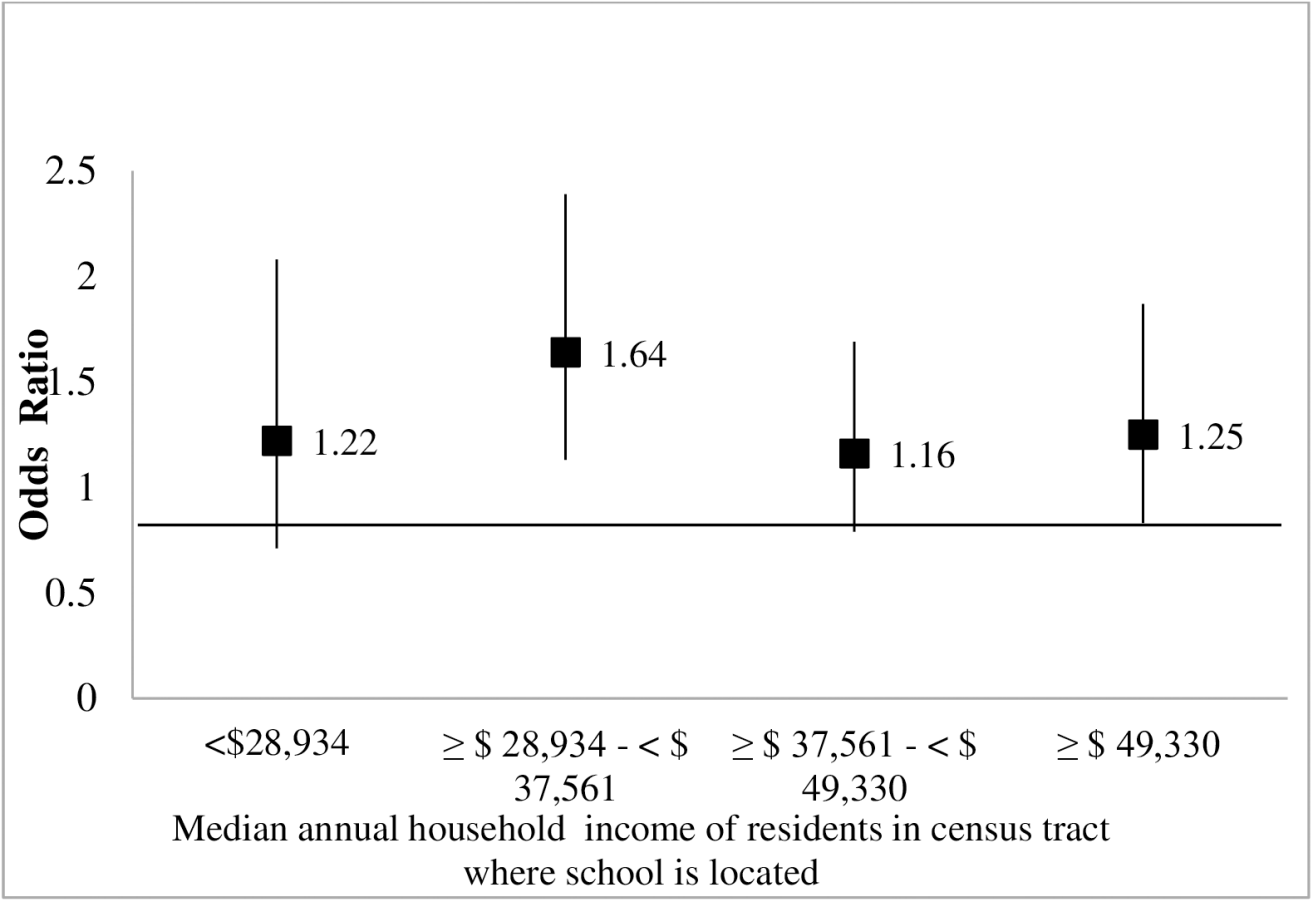
Associations ^a^ between PE policy compliance ^b^ and Latino children’s fitness, for each^c^ school neighborhood income category.^d,e^. ^a^Based on multilevel logistic regression models of the association between compliance with PE policies and children’s fitness, adjusted for age, gender, school enrollment, and proportion of children eligible for free or reduced priced meals, plus an interaction term between compliance and quartiles of (a) % of residents in schools census tract who have 16 or more years of education or (b) median annual household income. ^b^Compliance was measured at the district level and was defined as providing the minimum requirement for elementary-school students of 200 minutes of physical education every 10 days. ^c^For reference, the overall association between school district PE policy compliance and physical fitness, prior to including interactions was OR = 1.38 (95%CI: 1.07, 1.78). ^d^Modeled separately due to high correlation with residents’ education levels. ^e^Odds ratios obtained by combining the coefficient of the main effect of the policy and the coefficient for the interaction term between the policy main effect and the corresponding dummy variable for the education category. *Source*: Authors' analysis of data from the Census 2000 Summary File 3 and from the CBEDS School Information Form and California School Free or Reduced Meal Program (both publicly available on the California Department of Education website (http://www.cde.ca.gov/ds/sd/sd/ and http://www.cde.ca.gov/ds/sh/cw/filesafdc.asp), and data from the California Fitnessgram (2004–2005 and 2005–2006), California Department of Education.

**Fig 2:**
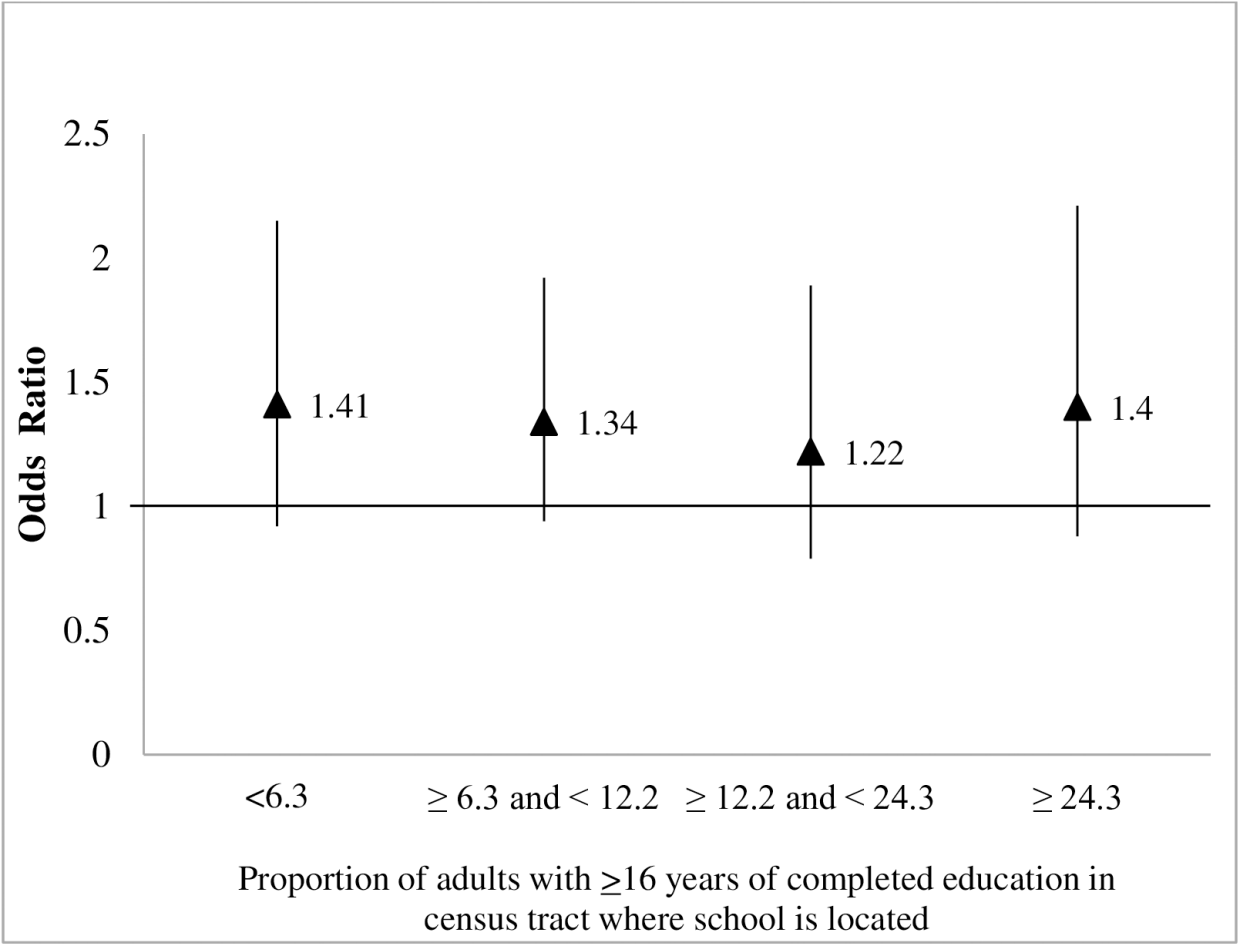
Associations^a^ between PE policy compliance^b^ and Latino children’s fitness, for each^c^ school neighborhood education category.^d,e^. ^a^Based on multilevel logistic regression models of the association between compliance with PE policies and children’s fitness, adjusted for age, gender, school enrollment, and proportion of children eligible for free or reduced priced meals, plus an interaction term between compliance and quartiles of % of residents in schools census tract who have 16 or more years of education. ^b^Compliance was measured at the district level and was defined as providing the minimum requirement for elementary-school students of 200 minutes of physical education every 10 days. ^c^For reference, the overall association between school district PE policy compliance and physical fitness, prior to including interactions was OR = 1.38 (95%CI: 1.07, 1.78). ^d^Modeled separately due to high correlation with annual household income. ^e^Odds ratios obtained by combining the coefficient of the main effect of the policy and the coefficient for the interaction term between the policy main effect and the corresponding dummy variable for the income category. *Source*: Authors' analysis of data from the Census 2000 Summary File 3 and from the CBEDS School Information Form and California School Free or Reduced Meal Program (both publicly available on the California Department of Education website (http://www.cde.ca.gov/ds/sd/sd/ and http://www.cde.ca.gov/ds/sh/cw/filesafdc.asp), and data from the California Fitnessgram (2004–2005 and 2005–2006), California Department of Education.

There was no evidence of a statistically significant interaction between district-level compliance with PE policy and either of the two school neighborhood socioeconomic measures (p = 0.46 for income and p = 0.95 for education). Regardless of school neighborhood income or education levels, Latino fifth-graders in compliant districts appeared more likely to meet or exceed fitness standards than those in noncompliant districts, after adjustment for other student-, school-, and district-level factors (Figs 1 and 2). Specifically, within each of the school neighborhood income or education quartiles, school district compliance with the PE policy was associated with greater likelihoods of physical fitness compared with children in districts that were found to be non-compliant with the PE policies; the associations were not statistically significant for most quartiles. However, the overall positive association between district compliance with physical education policy and fitness was OR = 1.38 (1.07, 1.78) adjusted for covariates and prior to including the interactions in the model.

## Discussion

The association between district-level compliance with state PE policy and physical fitness did not appear to vary significantly by the levels of income or education in neighborhoods in which schools were located. Although this finding does not reflect a causal association, it suggests that school district compliance with PE policies may have the potential to influence Latino children’s fitness patterns, irrespective of school neighborhood socioeconomic advantage. We also found that a substantial proportion (84%) of Latino fifth graders attended schools in districts that were reported to be noncompliant with the California state PE policy.

This study relied on secondary data, thus there are several limitations related to the cross-sectional nature of the data; the relatively small subset of school districts for which compliance data were available; the lack of school-specific information on the quality and quantity of PE; and other opportunities for physical activity and individual student characteristics. Cross-sectional data preclude causal inferences about the direction of the association between district-level compliance with PE policies and fitness levels among Latino fifth-graders.

Additionally, because the nominal variable for compliance could be obtained for only a small subset of school districts statewide and was not available at the school level, we could not, with greater precision, examine how actual provision of the minimum time of physical education in schools or districts attended by students was associated with compliance or noncompliance. It is possible that individual schools were in compliance with PE policies even in districts classified as noncompliant. Thus, we have likely underestimated the true association between PE policy compliance and physical fitness among Latino fifth-graders, although the effects of this misclassification bias on the potential moderating role of neighborhood income and education levels are difficult to predict.

We also had insufficient information to examine differences in student characteristics other than age and gender—such as acculturation, and generational status—that may have influenced fitness. We could not examine how district-level compliance related to the actual quantity and quality of PE in individual schools[29] and/or the availability of recreational opportunities and afterschool programs that might have influenced the observed differences in students’ physical fitness levels by district-level compliance.

Nevertheless, the present study contributes to our understanding of the potential influence of physical education policies on children’s fitness, by examining this association across levels of school neighborhood socioeconomic advantage and by using a large dataset of Latino students who attended public elementary schools in California. While the magnitude of the differences in the proportion of students who met or exceeded fitness standards (57.8% in policy-compliant districts vs. 55.6% in non-compliant districts) may not appear large, the numbers of children affected at the population level suggest important implications for health improvements early in life. The finding that regardless of the socioeconomic advantage of the school neighborhood, district-level compliance with PE policies was positively, though not always significantly, associated with Latino students’ physical fitness implies that compliance with PE policy may play a role in shaping Latino elementary students’ physical activity and fitness levels. Alternatively, the lack of a moderating effect of school neighborhood socioeconomic advantage may be due to limited variability in the socioeconomic distribution in neighborhoods in which these schools were located.

This study also found that a substantial proportion of Latino fifth-graders in this study attended schools in districts that were noncompliant with the state PE policy. This finding suggests that compliance with physical education policies should be a priority across all schools. Strong physical education mandates in schools, along with provisions for compliance [33], may play an important role in promoting children’s physical fitness, particularly for Latino children who are more likely to live in socioeconomically disadvantaged neighborhoods with relatively limited opportunities for physical activity [17,34] and less likely to participate in organized sports [35] than their non-Hispanic white peers. PE policies have the potential to significantly contribute to population-level improvements in children’s physical activity and fitness, especially in states such as California where Latino children represent 51% of the population younger than eighteen years [36]. The accuracy of race/ethnicity indicators is uncertain, though other fitnessgram indicators such as overweight/obesity show racial/ethnic patterns consistent with other California data.

The California law requires that elementary schools provide to their students a minimum of 200 minutes of PE every ten school days, which falls below the 150 minutes per week of elementary school physical education recommended by the Institute of Medicine [3,37]. Research has found that state and district policies requiring a specified number of weekly minutes of physical activity at school are significantly associated with greater likelihood of schools having 150 minutes of weekly PE [38]. Until recently, however, only a few states had PE policies requiring a specified number of minutes of physical activity at school; many of these policies were weakly worded, and monitoring for compliance is inadequate or nonexistent [19]. PE adherence could be improved if evidence is provided in regards to how PE policies can be achieved within the schools already existing structures [39] and with public disclosure of school PE compliance [40].

## Conclusion

This study’s findings suggest that attending schools where physical education programs are in compliance with state PE policies may provide important opportunities for promoting physical fitness among Latino elementary-age children in California, across neighborhoods with varying levels of socioeconomic advantage. Strategies to improve Latino student’s fitness and health should include efforts to monitor and encourage compliance with PE policies at all schools.
